# Fatty Acid 2-Hydroxylase and 2-Hydroxylated Sphingolipids: Metabolism and Function in Health and Diseases

**DOI:** 10.3390/ijms24054908

**Published:** 2023-03-03

**Authors:** Matthias Eckhardt

**Affiliations:** Institute of Biochemistry and Molecular Biology, Medical Faculty, University of Bonn, 53115 Bonn, Germany; eckhardt@uni-bonn.de

**Keywords:** cancer, fatty acid hydroxylase-associated neurodegeneration, fatty acid 2-hydroxylase, leukodystrophy, hereditary spastic paraplegia, myelin, neurodegeneration with brain iron accumulation, neurodegeneration, sphingolipids, skin

## Abstract

Sphingolipids containing acyl residues that are hydroxylated at C-2 are found in most, if not all, eukaryotes and certain bacteria. 2-hydroxylated sphingolipids are present in many organs and cell types, though they are especially abundant in myelin and skin. The enzyme fatty acid 2-hydroxylase (FA2H) is involved in the synthesis of many but not all 2-hydroxylated sphingolipids. Deficiency in FA2H causes a neurodegenerative disease known as hereditary spastic paraplegia 35 (HSP35/SPG35) or fatty acid hydroxylase-associated neurodegeneration (FAHN). FA2H likely also plays a role in other diseases. A low expression level of FA2H correlates with a poor prognosis in many cancers. This review presents an updated overview of the metabolism and function of 2-hydroxylated sphingolipids and the FA2H enzyme under physiological conditions and in diseases.

## 1. Introduction

**Figure 1:**
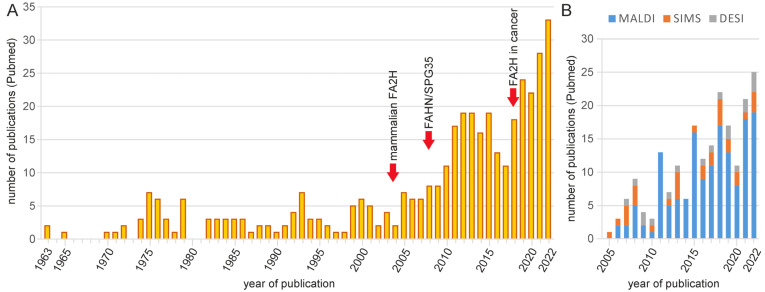
Number of publications dealing with 2hFA-SL and mass spectrometry imaging of sphingolipids listed in Pubmed. (**A**) Number of publications per year related to FA2H and 2-hydroxylated sphingolipids. The increase over the last 15 years was likely driven by the identification of the mammalian *FA2H* gene (in 2004/2005), the description of human FAHN/SPG35 disease (since 2008) and a likely role of FA2H in tumor progression in several cancer types (since 2018). Pubmed (https://pubmed.ncbi.nlm.nih.gov/; accessed on 14 February 2023) queries were performed using the following search terms: (2-hydroxyl* AND sphingo*), “2-hydroxy fatty acids”, “alpha-hydroxylated fatty”, FA2H, “fatty acid 2-hydroxylase”, “fatty acid alpha-hydroxylation”, “fatty acid hydroxylase-associated neurodegeneration” or SPG35. (**B**) Number of publications per year dealing with mass spectrometry imaging of sphingolipids (though not necessarily 2hFA-SL). The most widely used method is matrix-assisted laser desorption/ionization (MALDI), followed by time-of-flight secondary ion mass spectrometry (TOF-SIMS) and desorption electrospray ionization (DESI) imaging mass spectrometry. Pubmed query search terms: (MALDI OR SIMS OR DESI) AND imaging AND sphingo*.

Sphingolipids are important components of biological membranes that play multiple important roles in signal transduction, intracellular trafficking, apoptosis, cell differentiation and other processes [1]. These various functions are reflected in the structural diversity of sphingolipids. The different chain length of the acyl residue or the sphingoid base, the degree of saturation, diversities in the polar head group (particularly within the complex glycosphingolipids), hydroxylations of the sphingoid base and the acyl residue add structural diversity that appears to be important for the physiological function of sphingolipids. Hydroxylation can occur at C-4 of the sphingoid base and at C-2, C-3 and ω-C of the acyl residue [2]. This review only deals with the biosynthesis and function of sphingolipids with a 2-hydroxyl group in the acyl residue. The interest in these 2-hydroxylated fatty acids (2hFA) containing sphingolipids (2hFA-SL) has steadily increased over the last 15 years. This interest was stimulated by the characterization of the fatty acid 2-hydroxylase (FA2H) in mammals and other higher eukaryotes in 2004, which was later followed by the identification of a human disease (FAHN/SPG35) caused by mutations in the *FA2H* gene in 2008 (Figure 1A). The observation that FA2H expression level affects tumor progression in different cancer types further strengthened interest in the *FA2H* gene and 2hFA-SL.

2hFA and 2hFA-SL are found in all kingdoms of eukaryotes (Animalia, Plantae, Fungi and Protista) and in eubacteria. Though this review primarily discusses the role of 2hFA-SL in mammals and human diseases, several aspects of the synthesis and function of 2hFA-SL in invertebrates, plants, fungi and bacteria will not be ignored, as these may also provide some additional hints as to the functional role and synthesis of 2hFA-SL in mammals, which are currently not well understood.

2hFA-SL are especially abundant in some tissues, e.g., the brain, specifically in myelin, and skin. With the development of sensitive methods, however, 2hFA were identified in many tissues [3]. A comprehensive overview of the occurrence of 2hFA-SL in mammalian tissues can be found in the review by Hama [4]. Different tissues and cell types differ significantly with respect to the relative amount of 2hFA-SL, chain lengths of 2hFA and sphingolipid species that are 2-hydroxylated [4].

Currently, the only known enzyme that hydroxylates straight fatty acids at position 2 in eukaryotes is the FA2H enzyme in mammals or its orthologs in other classes. Two stereoisomers can be formed by the hydroxylation of acyl residues. In mammals (likely in all vertebrates and possibly in all eukaryotes), the (R)-enantiomer is synthesized [5]. It is assumed that the presence of the (S)-enantiomer in milk and brain samples from animals and also vegetable oils is derived originally from bacterial sources [6].

2hFA-SL are present in several bacteria, e.g., Flavobacterium and Sphingomonas species, and the relative abundance of 2hFA-SL differes significantly under different growth conditions [7,8]. Many bacteria synthesize the (S)-enantiomer [8,9]. Fatty acid 2-hydroxylases have been cloned from Sphingomonas species [10] and are not homologues of the eukaryotic FA2H but are cytochrome P450 (CYP450) hydroxylases acting in a H_2_O_2_-dependent manner [11,12]. In addition to these, there are several myxobacteria species that have functional fatty acid 2-hydroxylases with significant similarities to the eukaryotic FA2H enzyme, including conserved histidine motifs that form the active center of the enzyme [13]. Notably, the stereochemical specificity of these prokaryotic *FA2H* orthologs differs between species: while some add the hydroxyl group in the (S)-configuration, such as other bacteria using CYP450 enzymes, others synthesize (R)-2-hydroxy fatty acids, such as the eukaryotic FA2H enzyme [13].

## 2. Analytical Methods for 2hFA-SL and Other Sphingolipids

Analytical methods for the detection and quantification of 2hFA-SL (as well as technical challenges) are, in general, the same as for their non-hydroxylated counterparts. There is no specific extraction method to obtain only 2-hydroxylated sphingolipids because the extraction behavior is much more influenced by the (polar or anionic) head group of the lipid. Thus, non-hydroxylated and the corresponding 2-hydroxylated sphingolipids are analyzed together, and their molar ratio often provides valuable information. In most studies, lipids are extracted using one of the liquid–liquid biphasic systems composed of water and organic solvents (such as the Bligh Dyer [14] or Folch [15] method using methanol and chloroform, or chloroform-free methods using methyl-*tert*-butyl ether [16] or 1-butanol [17], or modifications of these [18]; see [19,20] for comprehensive reviews). Anionic sphingolipids (e.g., sphingosine-1-phosphate or sulfatide (see Section 5.2)) and complex glycosphingolipids require modifications or different extraction methods to ensure an efficient recovery of the lipids [19,20]. 

The current standard method for the structural analysis and quantification of 2hFA-SL (as well as non-2hFA-SL) in lipidomic studies, as well as in the analysis of individual lipids or lipid classes, is liquid chromatography–tandem mass spectrometry (LC-MS/MS). An overview of the workflow in mass spectrometry lipidomics is presented in the review by Köfeler et al. [21]. Although direct infusion mass spectrometry is possible, chromatography (reversed phase-HPLC or hydrophilic interaction liquid chromatography [22]) is usually applied because it provides additional information about the lipid structure and reduces the complexity of the sample. A comprehensive review of the specific requirements for the mass spectrometry of glycosphingolipids can be found in [23]. The C-2 position of the hydroxyl group in 2hFA-SL can usually be confirmed by a characteristic fragmentation pattern in the MS² spectrum [24]. The 2-hydroxyl group in hydroxylated-SL can cause problems of isobaric and isomeric interference because of the very similar mass of the hydroxylated lipid with the ^13^C_2_ isotope to a related lipid with a +1 chain length. This may be difficult to resolve by mass spectrometry and may require specific chromatography steps to separate the isobaric species [25]. Isomeric interference may be due to the identical mass of stereoisomers of sugars in glycosphingolipids (2-hydroxylated or not). The stereospecific discrimination between glucosylceramide and galactosylceramide was made possible by using a new hydrophilic interaction liquid chromatography–MS/MS protocol [26].

A very promising new approach in the area of spatial metabolomics is mass spectrometry imaging (MSI), which is also being progressively applied in (sphingo) lipid analysis [27,28,29,30]. Several studies performed MSI to detect 3’-sulfo-galactosylceramide (sulfatide) and 2hFA-sulfatide (which are abundant in myelin; see Section 5.2) and other sphingolipids in tissue sections using matrix-assisted laser desorption/ionization (MALDI)-MSI [31,32,33,34], time-of-flight secondary ion mass spectrometry (TOF-SIMS)-MSI [35,36,37] or desorption electrospray ionization (DESI)-MSI [38]. By combining the high spatial resolution of SIMS with the high mass resolution of the Orbitrap mass spectrometer in a method called 3D OrbiSIMS, Passarelli et al. [39] could map the distribution of 2hFA-sulfatide in mouse brains at a cellular to subcellular resolution. Using MALDI-MSI, it was possible to demonstrate the differential distribution of non-hydroxylated sulfatide and 2hFA-sulfatide in a human neocortex [34]. With TOF-SIMS-MSI, Hirahara et al. [40] demonstrated a sequential change from C18-2hFA-sulfatide in oligodendrocyte progenitor cells (OPC) to C20-2hFA-sulfatide in differentiating oligodendrocytes and C24-2hFA-sulfatide in mature oligodendrocytes. Nakashima et al. [41] demonstrated a differential localization of C22:0/C24:0-2hFA-sulfatide with phytosphingosine as a long chain base in intercalated mouse renal cells in combination with the mislocalization of vesicular H^+^-ATPase, suggesting a role of these 2hFA-sulfatide species in NH_4_^+^ and H_3_O^+^ excretion. It is obvious that the analysis of lipids in tissue samples by MSI with cellular and possibly subcellular resolution has outstanding potential for the analysis of functional roles of lipids, including 2hFA-SL.

## 3. Biosynthesis of hFA and hFA-SL

### 3.1. Fatty Acid 2-Hydroxylase (FA2H)

**Figure 2:**
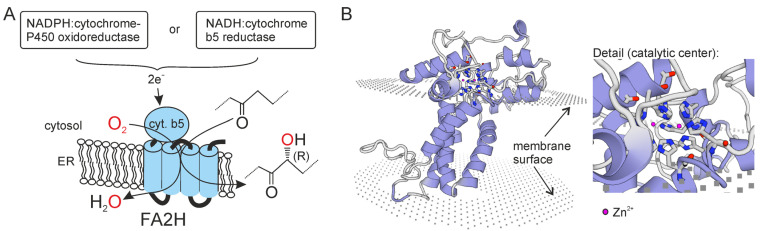
Enzymatic reaction catalyzed by FA2H and structure of the enzyme. (**A**) Reaction scheme of the 2-hydroxylation reaction of FA2H. (**B**) Model of the human FA2H enzyme (without cytochrome b5-like domain). Molecular modeling of human FA2H (Ac. Q7L5A8) was performed using Swiss Model (https://swissmodel.expasy.org/; accessed on 14 January 2023) with yeast FA2H (SCS7p; PDB file AZR0) as template. The histidine residues and the two zinc ions forming the catalytic center of the hydroxylase domain of the enzyme are highlighted and shown in the enlarged section on the right.

The fatty acid 2-hydroxylase enzyme, which is encoded by the *FA2H* gene in humans, has orthologs in apparently all eukaryotes. The *FA2H* gene has been characterized in mammals [42,43], yeast (*SCS7*) [44,45], plants (*FAH1*, *FAH2*) [46] and protists [47]. The FA2H enzyme belongs to the fatty acid hydroxylase/desaturase gene family and is an NAD(P)H-dependent monooxygenase that localizes to the endoplasmic reticulum [43]. The catalytic center is composed of four conserved histidine motifs that form an essential di-metal ion center in the catalytic center of the enzyme [48] (Figure 2). The enzyme adds the hydroxyl group in a stereospecific manner and forms only the (R)-enantiomer [49]. While in most eukaryotic genomes only one *FA2H* gene is present, *Arabidopsis thaliana* and other plants express two related *FA2H* genes, *FAH1* and *FAH2* [50], which differ with respect to their substrate specificity. The FAH1 enzyme mainly uses mainly very long chain fatty acids (VLCFAs) as substrates, whereas FAH2 prefers long chain fatty acids (LCFAs) [51]. In animals, fungi and protists, the FA2H enzyme contains an N-terminal cytochrome b5-like domain that is responsible for electron transfer from NAD(P)H. In contrast, the plant enzymes lack this domain but interact with one of the separate cytochrome b5 proteins within the ER membrane [52]. Whether cytochrome b5 can partially functionally replace the cytochrome b5-like domain, e.g., in cases of FAHN (see Section 6), with mutations in this domain is not known.

The X-ray crystal structure of the baker’s yeast FA2H enzyme (SCS7p) has been resolved, though without the N-terminal cytochrome b5-like domain [48]. This study confirmed the previously predicted four-transmembrane domain structure [42,43] of the enzyme, with N-terminal cytochrome b5 domain and C-terminus facing the cytosol. Although FA2H is regarded as a di-iron enzyme, the yeast ortholog contains two zinc ions in the di-metal ion binding site [48]. Based on structural differences between SCS7p and the functionally and structurally related stearoyl-CoA desaturase-1 (SCD1), it was concluded that acyl-CoAs are most likely not substrates for FA2H/Scs7p, whereas a ceramide fits well into the catalytic center of the enzyme. On the other hand, the only established in vitro enzymatic FA2H assay used free fatty acids as an efficient substrate [53]. It is an open question whether or to what extent free fatty acids may serve as substrates in vivo.

Heme is synthesized in mitochondria and thus must be transferred to the ER-localized FA2H protein when its cytochrome b5-like domain is folded into its native conformation. A screen for FA2H interaction partners identified progesterone receptor membrane component 1 (PGRMC1) as a binding partner of FA2H [54]. PGRMC1 binds heme and is a putative heme chaperon [55]. Its yeast homologue (Dap1) is known to be required for the activation of several CYP450 enzymes [56]. A PGRMC1 antagonist reduced FA2H activity [54], and PGRMC1 may be involved in the delivery of heme to the cytochrome b5 domain of FA2H.

### 3.2. Alternative Pathways of 2hFA Synthesis

**Figure 3:**
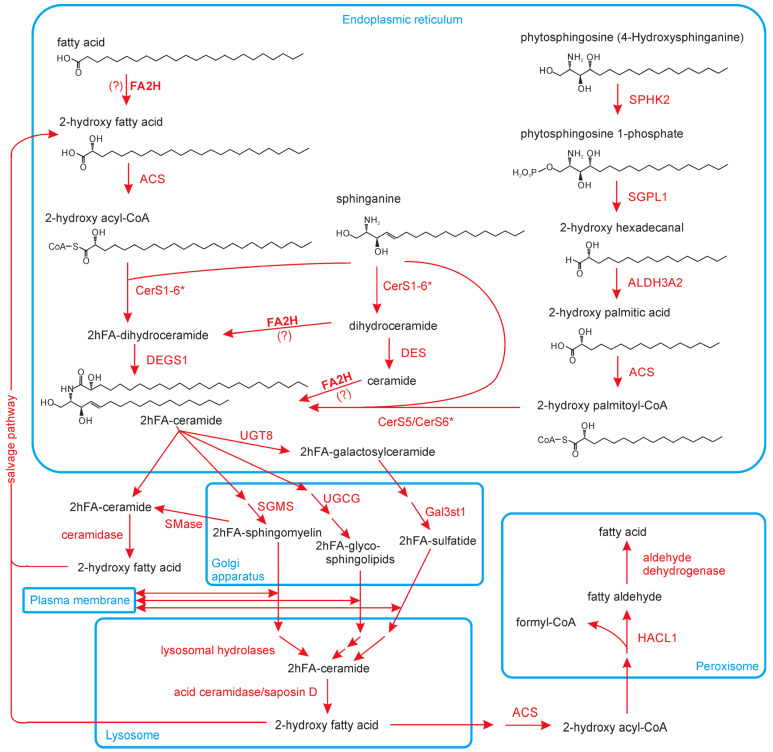
Overview of the metabolism of 2hFA-SL. As the physiological substrate(s) of FA2H is/are not yet clear, FA2H-dependent reactions are found here at different steps of the pathway, and the reactions are labeled with question marks (?) to indicate this fact. CerS1-6*/CerS5/CerS6*: In mammals, all six known ceramide synthases (CerS1 to CerS6) are capable of using 2-hFA-CoA as substrate. However, only CerS5 and CerS6 accept C16 acyl-CoA [63]. The FA2H-dependent reactions are shown with a C24 fatty acid/acyl residue; however, the enzyme apparently accepts acyl residues or fatty acids of various chain lengths. The α-oxidation of phytosphingosine can also metabolize longer sphingoid bases and thus generate longer 2hFA [61]. Note that many reactions, enzymes and metabolites, especially in the Golgi apparatus and lysosomes, are not specified in this Figure. Abbreviations/gene names and Enzyme Commission numbers: ACS—acyl-CoA synthetase (EC 6.2.1.2./6.2.1.3.); ALDH3A2—aldehyde dehydrogenase 3A2 (EC 1.2.1.3); (acid) ceramidase (EC 3.5.1.23); CerS(1-6)—ceramide synthase 1 to 6 (EC 2.3.1.24); DEGS1—sphingolipid-4-desaturase (EC 1.14.19.17); FA2H—fatty acid 2-hydroxylase (EC 1.14.18.6); Gal3st1—galactose-3-O-sulfotransferase 1 (EC 2.8.2.11); HACL1—2-hydroxylacyl-CoA lyase 1 (EC 4.1.2.63); SGMS—sphingomyelin synthase (EC 2.7.8.27); SGPL1—sphingosine-1-phosphate lyase 1 (EC 4.1.2.27); SMase—sphingomyelin phosphodiesterase (EC 3.1.4.12); SPHK2—sphingosine kinase 2 (EC 2.7.1.91); UGCG—UDP-glucose:ceramide glucosyltransferase (EC 2.4.1.80); UGT8—UDP-galactose:ceramide galactosyltransferase (EC 2.4.1.47).

FA2H is the most studied enzyme involved in the synthesis of 2hFA in eukaryotes. However, the FA2H enzyme is not the only enzyme capable of synthesizing 2hFA or 2hFA-ceramides. It is clear that mice lacking a functional *Fa2h* gene seem to be devoid of 2hFA-SL in the nervous system [57,58] yet still contain 2hFA-SL in various organs, such as the skin [59]. In addition, levels of 2hFA-sphingomyelin in lymphocytes and erythrocytes from FAHN/SPG35 patients with a mutation causing exon 5/6 skipping (which is expected to fully abolish FA2H activity) were not reduced [60]. One alternative source for 2hFA is an α-oxidation pathway in the ER [61]. Through this pathway, 2-hydroxylated palmitic acid can be formed from phytosphingosine (Figure 3). However, this pathway mainly generates C16-2hFA (and C18-2hFA from C20-phytosphingosine base). Longer phytosphingosine bases that could potentially enable the synthesis of VLCFA 2hFA (>C_20_) are rare. As *FA2H* knockout mice and FAHN patients still contain substantial amounts of VLCFA 2hFA-SL [59,60], it is very likely that additional enzymes, which have not yet been characterized, exist that are capable of synthesizing 2hFA/2hFA-SL, at least in mammals. The only other known mammalian fatty acid 2-hydroxylase, peroxisomal phytanoyl-CoA hydroxylase, appears to be unable to hydroxylate straight fatty acids. As CYP450 enzymes synthesize 2hFA in certain bacteria (see above), they are possible candidates for these currently unknown enzymes.

### 3.3. Degradation of 2hFA-SL

The degradation of 2hFA-SL occurs mainly in lysosomes (Figure 3). This is achieved by the same acid hydrolases that degrade their non-hydroxylated counterparts. The sphingolipid activator protein (saposin) D, one essential cofactor of acid ceramidase, seems to be mainly involved in extracting the 2hFA-ceramide from the membrane to enable its hydrolysis [62]. Alternatively, the amide bond of 2hFA-ceramide may be hydrolyzed by alkaline or neutral ceramidase outside the lysosome; however, to what extent this happens with the 2hFA-ceramide is unclear. The released 2hFA can likely be recycled through a salvage pathway (Figure 3). All six mammalian ceramide synthases (CerS1-6) accept 2hFA-CoA as substrate [63]. Alternatively, 2hFA can be degraded through peroxisomal α-oxidation. In addition to branched chain fatty acids, peroxisomal 2-hydroxyphytanoyl-CoA lyase is able to cleave 2-hydroxylated straight chain fatty acids [64]. Whether free 2hFA have a physiological function is currently not known. However, exogenously added 2hFA can dramatically affect cell physiology (see Section 6).

## 4. 2hFA-SL in Membrane Trafficking and Microdomains

What is the functional role of 2hFA-SL in biological membranes? Knockout and knock-down experiments as well as biophysical studies in artificial membranes have been performed to address this question. The results suggest that 2hFA-SL has a significant influence on the structure and stability of membrane microdomains and affects the trafficking of membranes/membrane proteins. Support for a functional role of 2hFA-SL in membrane trafficking comes from studies in *Drosophila melanogaster*, which demonstrated that *FA2H* overexpression can interfere (depending on the genetic background) with the apical membrane trafficking of newly synthesized Rhodopsin via Rab11-positive vesicles (recycling endosomes) to the rhabdomeres [65]. Along this line, monitoring the localization of 2hFA-SL in *Caenorhabditis elegans* (using 2-hydroxy palmitic acid alkyne and click chemistry labeling with azide-Cy3) revealed its accumulation in the apical membrane of polarized cells in the intestinal tract [66]. The loss of the *C. elegans FA2H* gene *fath-1* strongly affected distribution of late and recycling endosomes, which then accumulated in the apical region of intestinal cells [66]. The inactivation of fath-1 strongly reduced the level of C17:1^Δ10^ (heptadecenoic acid) that can be generated through the α-oxidation of 2hFA-C18:1. This indicates the possibility that the effects of FA2H effects are not necessarily mediated by 2hFA or 2hFA-SL but may also depend on downstream reaction products. An important role of 2hFA-SL in apical membrane sorting was also suggested by the observation that the polarization of MDCK cells is associated with a strong increase in the percentage of hydroxylated sphingolipids [67], though this study did not discriminate between different hydroxylations.

Hydroxyl groups of the sphingoid base are essential for lipid–lipid interactions in the membrane through the formation of inter- and intramolecular hydrogen bonds [68]. Likewise, the 2-hydroxyl group of the fatty acid residue plays a comparable role in stabilizing lipid–lipid interactions. The 2-hydroxyl group appears to stabilize membrane microdomains, possibly through the additional intermolecular hydrogen bonds between the 2-hydroxyl group, the amino group of the sphingosine and the sugar head group [69,70,71]. Glycosyl inositol phosphorylceramides (GIPCs) are abundant sphingolipids in the tobacco (*Nicotiana tabacum*) cell plasma membrane (comprising up to 80% of the lipid molecules in the outer leaflet). It was shown that GIPCs containing 2hFA are specifically enriched in detergent-insoluble membranes that form small membrane domains and interact with phytosterols within membranes via hydrogen bonds [72,73]. In line with this, plant cells lacking *FAH1* and *FAH2* have reduced levels of membrane microdomains [74]. In artificial membranes, the size of the membrane domains containing 2hFA-galactosylceramides was smaller, but they covered twice the membrane when compared to membranes containing non-hydroxylated galactosylceramide [75].

The reduced expression of *FA2H* in adipocytes increased the mobility of raft-associated lipids and thereby reduced the plasma membrane levels of insulin receptors and GLUT4 glucose transporters [49,76]. This effect was abolished specifically by the addition of ®-2-hydroxy palmitic acid (but not the (S)-stereoisomer), which was incorporated into hexosylceramides. Such a stereo-specific effect was also observed for the antiproliferative activity of synthetic 2hFA-ceramides in which the (R)-enantiomers had a much stronger activity [69]. Such enantiomer-specific effects should result from stereospecific interactions, which could occur through a specific protein (this possibility has, however, not been reported yet) or a stereo-specific interaction with another lipid or a functional group within the same sphingolipid molecule (e.g., with the glucose or galactose residue). For example, while 2hFA-sphingomyelin stabilizes, 2hFA-phytosphingomyelin (containing an additional hydroxyl group at C-4 of the sphingoid base; see Figure 3) destabilizes the gel phase of a membrane [77].

## 5. FA2H Expression and Function in Different Tissues

### 5.1. FA2H Expression Levels in Mammalian Tissues and Different Cell Types

**Figure 4:**
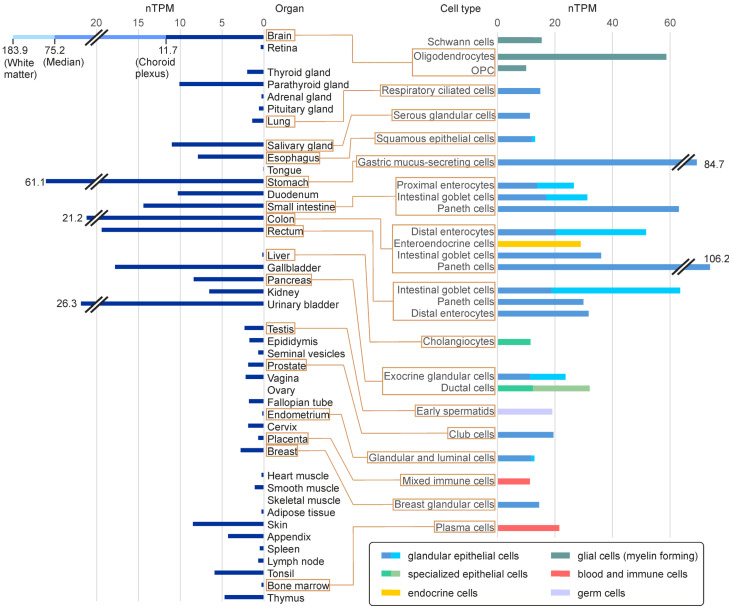
*FA2H* expression levels in human organs and selected cell types with high *FA2H* expression levels. Bars indicate the mean normalized expression (TPM = transcripts per million) derived from RNA sequencing data that are accessible via the Human Protein Atlas (https://www.proteinatlas.org/; accessed on 14 February 2023) [80]. For the brain, only the median expression over all brain regions and the maximal (found in white matter) and minimal (in choroid plexus) expression levels are shown. Expression levels in individual cell types were taken from the Human Protein Atlas datasets of single-cell type transcriptomes [81]. Only cell types with a normalized expression level > 10 nTPM are displayed. For cell types where several expression levels were reported in the data base, the range is depicted (dark bar = lowest; light bar = highest expression level). Abbreviation: OPC—oligodendrocyte progenitor cell.

*FA2H* expression is detectable in most tissues, though its level varies strongly between different organs/tissues and cell types (Figure 4). In humans, *FA2H* mRNA is only undetectable in the ovaries and skeletal muscle. In addition to the brain and peripheral nervous system (PNS), where myelinating cells (oligodendrocytes and Schwann cells, respectively) express *FA2H*, it is particularly abundant in the intestinal tract. Looking at individual cell types, strong *FA2H* expression is particularly present in glandular epithelial cells (Figure 4, right panel). Accordingly, *FA2H* expression in skin is mainly found in sebaceous glands [78]. A high-level expression in glandular epithelial cells was also observed in murine tissues [59,79]. With the exception of sebocytes (see Section 5.3), there is currently no knowledge about the role of FA2H or 2hFA-SL in glandular epithelial cells. Unfortunately, there are almost no reliable data on the FA2H protein level in different tissues because of a lack of specific and sensitive antibodies.

### 5.2. FA2H in the Nervous System

**Figure 5:**
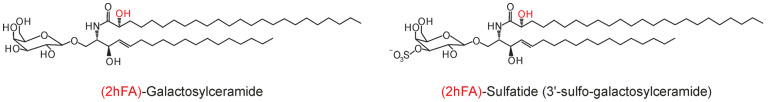
Structure of galactosylceramides and sulfatides (3’-sulfo-galactosylceramide). Both sphingolipids are present as non-hydroxylated and 2-hydroxylated (> 50% in mammals) variants in the myelin of the brain and PNS in most (though not all) vertebrates.

In the mammalian nervous system (and in many non-mammalian vertebrates), the majority of 2hFA-SL are 2hFA-galactosylceramide and 2hFA-sulfatide, which are abundant components of the myelin sheath [82] (Figure 5). It is, however, worth mentioning that some vertebrates do not have 2hFA-SL in their myelin [83]. *FA2H* is highly expressed in the myelinating cells (oligodendrocytes in brain and Schwann cells in PNS) [42,43]. An analysis of two independent *FA2H*-deficient mouse lines, generated by gene targeting, confirmed the assumption that the synthesis of these 2hFA-SL in the brain and PNS fully depends on FA2H activity [57,58]. Nonetheless, myelin was normally formed in these mice. However, adult mice developed a late-onset demyelination and axonal degeneration with motor behavioral deficits that are reminiscent of human FAHN/SPG35 disease (see Section 6.1). Thus, 2hFA-SL are not essential structural components of myelin that are necessary to build up compact myelin sheaths to enable saltatory nerve conduction, but they are required for long-term myelin maintenance and axonal support. Galactosylceramides and sulfatides have important functions in the differentiation of oligodendrocytes and the establishment of stable paranodal junctions at the node of Ranvier [82,84,85,86]. These are, however, apparently normal in *FA2H*-deficient mice. Thus, sulfatides and galactosylceramides do not have to be 2-hydroxylated to fulfill these functions.

### 5.3. 2hFA-SL and FA2H in the Skin

Skin contains large amounts of 2-hydroxylated and ω-hydroxylated ceramides and glucosylceramides [2,87]. While the essential role of the ω-hydroxylation for the formation of the permeability barrier is well known, the specific role of the 2-hydroxylation is not clear. *FA2H* expression is upregulated during in the vitro differentiation of human keratinocytes, and 2hFA-ceramides/glucosylceramides appear to be required for the formation of epidermal lamellar membranes that are essential to build up the epidermal permeability barrier [88]. In *FA2H*-deficient mice, however, the 2hFA-ceramide level in skin was unaltered and only a fraction of the 2hFA-glucosylceramide level (acyl chain length C20 to C24) was reduced [59]. Moreover, the permeability barrier was apparently unaffected, in line with undetectable *FA2H* expression in keratinocytes. The possibility that FA2H plays different roles in human and mouse keratinocytes remains to be determined.

Skin is also the only organ where the role of FA2H in glandular epithelial cells has been examined to some extent. *FA2H* is strongly expressed in sebocytes, and FA2H-deficient mice have enlarged sebaceous glands and develop a cyclic alopecia [59]; the latter is possibly a consequence of altered sebum composition with a strongly reduced wax diester level that causes a significant increase in the melting temperature of the sebum above body temperature [59,79]. A chemically induced *FA2H* mouse mutant (‘sparse’) exhibited a comparable phenotype [89]. Wax diester synthesis requires 2hFA [90]. However, because 2hFA may also be formed by FA2H independently (see Section 3.2), it is unclear whether FA2H is directly involved in this reaction. Interestingly, mice deficient in the ceramide synthase CerS4 (the major ceramide synthase in sebaceous glands) and alkaline ceramidase 1 (ACER1)-deficient mice exhibit a very similar skin/sebaceous gland phenotype [91,92]. This may suggest a critical role of 2hFA-ceramide/2hFA-SL turnover in sebocytes. A significant role of FA2H in mammalian skin physiology is further supported by an *FA2H* mutation causing ichthyosis congenita in a Chianina cattle [93].

## 6. FA2H, 2hFA-SL and 2hFA in Human Diseases

### 6.1. Fatty Acid Hydroxylase-Associated Neurodegeneration (FAHN)

Deficiency in *FA2H* causes a human disease known as fatty acid hydroxylase-associated neurodegeneration (FAHN) or hereditary spastic paraplegia (HSP) type 35/spastic paraplegia 35 (HSP35 or SPG35) [94,95]. The disease is an autosomal recessive disorder belonging to the complex HSP type. It is a rare form among all hereditary spastic paraplegias known today [96,97]. Characteristic clinical signs in FAHN are spasticity of the lower limbs, ataxia, cognitive problems, and leukodystrophy [98]. FAHN is part of a group of diseases designated as neurodegeneration with brain iron accumulation (NBIA) [99]. Iron deposits in the basal ganglia that cause lesions in the globus pallidus (“eye-of-the-tiger sign”) are a typical finding in these diseases. Currently, more than 60 different mutations in the human *FA2H* gene that cause FAHN/SPG35 have been described in the literature [94,95,99,100,101,102,103,104,105,106,107,108,109,110,111,112]. Detailed description of the clinical signs and mutations identified will not be reviewed here, and the interested reader is referred to previous reviews/overviews on the disease [98,110].

Transgenic mouse lines with total or conditional *FA2H* knockout have been established and serve as animal models of FAHN [58,59]. Analysis of these mice showed that the synthesis of 2hFA-SL in myelinating cells is important for the maintenance of the myelin sheath, though not for myelin formation per se [58,59]. Phenotypes of both the total and the oligodendrocyte conditional *FA2H* knockout were similar and reminiscent of the symptoms of the human disease [58,59], indicating that most symptoms are caused by a deficiency of *FA2H* in the myelinating glia. Notably, however, the oligodendrocyte-specific *FA2H* knockout was not associated with learning and memory deficits, which were observed in the total knockout [59]. This suggests a (low) expression of *FA2H* in other cell types within the brain as well. It may thus be possible that cognitive impairment in FAHN could be a result of loss of FA2H activity in neurons. Nevertheless, most FAHN-related symptoms in the mouse models depend on *FA2H* loss in oligodendrocytes, leading to the question of how changes in the lipid composition of the myelin sheath cause axonal degeneration. A proteome analysis of myelin purified from *FA2H*-deficient mice found a relative specific accumulation of a major myelin protein called Opalin in compact myelin [113]. As the compact myelin corresponds to the apical membrane in polarized cells, this finding further supports the proposed role of 2hFA-SL in apical membrane sorting, as discussed previously. Altered trafficking or accumulation of myelin membrane proteins could be a possible link between altered myelin lipids and axonal pathology.

As mentioned above, FAHN belongs to a group of diseases known as neurodegeneration with brain iron accumulation (NBIA) that demonstrate a characteristic accumulation of iron in the basal ganglia [114], which has been observed in most FAHN patients [110]. However, how *FA2H* deficiency leads to brain iron accumulation is not understood. Fibroblasts from FAHN and other NBIA cases cultured in the presence of high iron exhibited a stronger intracellular iron increase compared to control fibroblasts. This possibly results from reduced lysosomal degradation and increased recycling of the transferrin receptor [115]. Mitochondrial dysregulation occurs in several types of NBIA [116]. Changes in mitochondrial fission and fusion altered the mitochondrial network in a *Drosophila* FAHN model [117]. This was accompanied by increased levels of the autophagy marker LC3 and its activated, lipidated form LC3-II, which was also observed in FAHN patient fibroblasts [117].

Although a hallmark of FAHN is the degeneration of upper motor neuron axons in the CNS, peripheral neuropathy has been observed in several FAHN patients [100,110,118]. A related, late-onset peripheral neuropathy was observed in older *FA2H*-deficient mice [58]. A proteome analysis of peripheral myelin identified elevated levels of a membrane complex containing the adhesion molecule CADM4, which is localized in Schmidt–Lanterman incisures [119]. This suggests that 2hFA-galactosylceramide or 2hFA-sulfatide may play a role in the trafficking or turnover of CADM4, though further studies are required to prove this.

### 6.2. Altered 2hFA-SL Levels or FA2H Expression in Other Diseases

Sphingolipids play important roles in many neurodegenerative diseases, such as Alzheimer’s (AD) or Parkinson’s disease (PD) [120]. Until now, however, there was no evidence that the 2-hydroxylation of sphingolipids or the expression of the *FA2H* gene had a significant influence in these or other neurodegenerative diseases. A strongly reduced brain sulfatide concentration was identified as an early event in AD [121,122], but there was no alteration of the 2hFA-sulfatide to total sulfatide ratio [34]. A genetic interaction of *PARK2* (mutated in early-onset PD) and *FA2H* mutations was reported by Benger et al. [108]. However, to the knowledge of the author of this review, there is currently no evidence for altered *FA2H* expression or 2hFA-SL levels in PD.

In a mouse model of Sjögren–Larsson syndrome, which is caused by mutations in the fatty aldehyde dehydrogenase gene *ALDH3A2* (see Figure 3), the brain concentration of 2hFA-galactosylceramide was significantly reduced. This may play a functional role in the disease [123]. Previously, it was shown that reduced 2hFA-galactosylceramide levels in transgenic mice destabilize the CNS myelin [124]. Notably, *ALDH3A2* strongly increased the activity of co-expressed *FA2H* (in CHO cells), suggesting that the ALDH3A2 substrate trans-2-hexadecenal (formed by degradation of sphingosine) may inhibit the FA2H enzyme (a reaction of the fatty aldehyde with a histidine residue in the catalytic center of the enzyme was proposed) [123]. The close proximity of the two enzymes ALDH3A2 and FA2H was suggested by the identification of ALDH3A2 as an interaction partner of FA2H [55], which would enable the efficient protection of FA2H by ALDH3A2.

FA2H may be a factor involved in obesity-induced insulin resistance as *FA2H* downregulation through the micro-RNA miR-3075 led to enhanced insulin signaling [125]. In early-onset obesity, miR-3075 is released via exosomes from hepatocytes and mediates enhanced insulin sensitivity. However, Guo et al. [49,76] found a downregulation of the insulin receptor upon *FA2H* knockdown. Thus, further studies are needed to clarify the role of FA2H in insulin signaling and resistance.

In many FAHN patients, bristly hairs with plaques attached to the hair shaft were observed [110]. This is, to some extent, reminiscent to the phenotype of *FA2H*-deficient mice [59]. As human sebum lacks wax diesters, this strongly suggests that, in addition to its possible role in sebum (wax diester) synthesis, FA2H or its reaction products have other important roles in sebocytes and/or keratinocytes. It is thus possible that FA2H may play a role in dermatological diseases.

### 6.3. FA2H and 2hFA-SL in Cancer

It is well known that sphingolipids have important functions in cancer cell signaling, cancer therapy and various aspects of tumor biology [126,127]. Therefore, it may not come as a surprise that changes in the abundance of 2hFA-SL in tumor cells have been observed in tumors of different origins [128,129,130]. High 2hFA-SL levels in carcinoma cells correlated with drug resistance [131,132]. In cases of cholangiocarcinoma, a lower survival rate of patients correlated with a higher relative abundance of 2-hydroxylated lactosylceramide (d18:1-h23:0/23:0) [133]. A strong association between high levels of 2hFA-hexosylceramides (most likely galactosylceramide) and high *FA2H* gene expression were found in low- and high-grade lung adenocarcinoma but not other lung cancers [134]. The latter study is a rare exception as it examined the 2hFA-SL levels together with *FA2H* expression.

A different picture emerged in more recent studies, which examined *FA2H* expression but not 2hFA-SL levels in various cancer types. *FA2H* knockdown in D6P2T Schwannoma cells stimulated cell proliferation by inhibiting cAMP-induced cell cycle arrest [135]. Further studies revealed an association between the *FA2H* expression level and tumor growth in different cancers, e.g., breast cancers, prostate cancer, gastric cancer and colorectal cancer: a low *FA2H* expression was associated with a reduced (disease-free) survival [136], reduced sensitivity against chemotherapeutic drugs [137] and, in general, a poor prognosis [138,139,140]. The over-expression of *FA2H* in tumor cell lines decreased cell proliferation, induced apoptosis and inhibited the epithelial–mesenchymal transition-associated gene expression [141,142]. Reduced tumor sensitivity towards the chemotherapeutic drug cisplatin [137] could be reversed by *FA2H* over-expression or treatment with (R)-2-hydroxypalmitic acid. Similarly. sensitivity to the drug PM02734 is significantly enhanced in tumor cells that over-express *FA2H* [143].

Taking all the studies together, we can observe increased malignancies with low *FA2H* expression on one side and with high levels of 2hFA-SL on the other side. One possible explanation for this apparent contradiction could be that increased levels of hFA-SL in many tumors depend on the not-yet-identified alternative 2-hydroxylase enzyme(s) mentioned above or the α-oxidation of phytosphingosine (Section 3), which could also have different substrate specificities. Since cytochrome P450 enzymes are often upregulated in tumor cells to confer drug resistance, some of them could potentially be involved in fatty acid 2-hydroxylation. Moreover, the 2-hydroxyl group may also have different effects depending on which sphingolipids are synthesized by a given tumor cell.

Different signaling pathways affected by *FA2H* over-expression have been identified: a higher chemosensitivity in gastric cancer cells depends on the inhibition of the mTOR/S6K1/Gli1 pathway by FA2H (through activation of AMPK) [137]; FA2H suppresses cancer stemness by inhibiting STAT3 and NF-κB signaling (through reduced phosphorylation of STAT3 and NF-κB) [144] and FA2H reduces metastasis in colon cancer through phosphorylation and the cytosolic retention of the transcription factor YAP1 [140]. Through which mechanisms FA2H influences these signaling pathways is, however, unclear at present.

Not much is known about the transcriptional regulation of the *FA2H* gene. In a breast cancer cell line, peroxisome-proliferator-activated receptor (PPAR)-α mediates the induction of FA2H by Δ(9)-tetrahydrocannabinol [145,146,147]. Zhou et al. [148] provided evidence that TNF-α, via the upregulation of *FOXC2*, increases *FA2H* expression in esophageal cancer. Several micro-RNAs target the human *FA2H* gene and may play important roles in its regulation in cancer or other diseases that have been identified [125,141,142,149].

2-hydroxylated oleic acid (2OHOA, Minerval) is an anti-cancer drug that is able to induce apoptosis in various cancer cells [150]. Free 2-hydroxylated fatty acids insert into membranes and significantly affect the membrane structure by reducing the lipid order and interfering with the tight packing of lipid side chains [151]. It is assumed that free 2OHOA affects signaling pathways in this way [152]. 2OHOA disturbs mitochondrial function by the uncoupling of oxidative phosphorylation and mitochondrial fragmentation [153]. Exogenously supplied 2OHOA is incorporated into triglycerides, diacylglycerides and phosphoglycerolipids [154,155]. However, there is no report describing the incorporation of 2OHOA into sphingolipids. It is therefore unclear whether the anti-tumor and pro-apoptotic pathways activated by 2OHOA treatment and *FA2H* overexpression are related.

## 7. Conclusions

Sphingolipids containing 2-hydroxylated acyl residues are present in many (if not all) cell types and tissues. While in many cases high levels of 2hFA-SL correlate with a high expression of *FA2H*, there is clear evidence that additional fatty acid 2-hydroxylase enzyme(s) exist. This or these currently unknown enzyme(s) and FA2H and their reaction products may, in part, fulfill opposing roles, as suggested by the analysis of tumor cells. They may also be potential targets in tumor therapy. Thus, an important task in the field of 2hFA-SL is the identification of this/these hydroxylases. In parallel, it must be established to what extent 2hFA synthesis occurs via the α-oxidation pathway in the endoplasmic reticulum. Given their abundance and important roles in different cellular processes and the important roles of sphingolipids in many diseases, alterations in the 2-hydroxylation of sphingolipids may be relevant in more diseases than currently anticipated.

## Data Availability

Data are available on request from the author.
